# Effects of semi-solidification of enteral nutrients on the pharmacokinetic behavior of orally administered carbamazepine in rats

**DOI:** 10.7150/ijms.35471

**Published:** 2019-09-07

**Authors:** Katsuhito Nagai, Sachiko Omotani, Masahito Shibano, Akihide Kobayashi, Akihiko Ito, Ikumi Nishimura, Yasutoshi Hatsuda, Junji Mukai, Hitomi Teramachi, Michiaki Myotoku

**Affiliations:** 1 Laboratory of Practical Pharmacy and Pharmaceutical Care, Faculty of Pharmacy, Osaka Ohtani University, 3-11-1 Nishikiori-kita, Tondabayashi, 584-0066, Japan; 2 National Hospital Organization Higashi-Ohmi General Medical Center, 255, Gochi-cho, Higashiomi, 527-8505, Japan; 3 Gifu Pharmaceutical University, 1-25-4 Daigaku-nishi, Gifushi, 501-1196, Japan

**Keywords:** Nutrition management, semi-solid enteral nutrient, carbamazepine, pharmacokinetics

## Abstract

The use of semi-solid enteral nutrients plays an extremely important role in accurate nutrition management. In the present study, we compared the pharmacokinetic profile of orally administered carbamazepine (CBZ) in rats treated with liquid RACOL^®^, semi-solid RACOL^®^, and HINE E-gel^®^, which are enteral nutrients marketed in Japan. Since liquid and semi-solid formulations are both marketed in Japan for RACOL^®^, liquid RACOL^®^ was orally administered to control rats. The serum concentration of CBZ at each sampling point was lower in the semi-solid RACOL^®^-treated group than in the liquid RACOL^®^-treated group. No significant differences were observed in the pharmacokinetic behavior of CBZ between the semi-solid RACOL^®^-treated and HINE E-gel^®^-treated groups. Regarding pharmacokinetic parameters, the impact of the area under the curve (AUC_0→5h_) was the liquid RACOL^®^ group > the semi-solid RACOL^®^ group ≈ the HINE E-gel^®^ group. Therefore, we concluded that serum concentrations of CBZ were lower when concurrently treating with semi-solid enteral nutrients than when simultaneously processing liquid enteral nutrients.

## Introduction

Adequate feeding with liquid enteral nutrients is essential for critically ill patients and helps to prevent malnutrition and its accompanying complications 1. Despite its benefits, feeding with liquid enteral nutrients is occasionally accompanied by adverse effects, such as diarrhea and aspiration pneumonia 2,3. Semi-solid enteral nutrients are considered to be useful for decreasing the adverse events associated with liquid enteral nutrients during feeding 4,5. However, dietary fibers used for the semi-solidification of enteral nutrients have been shown to interact with clinical drugs 6. We also previously demonstrated that the pharmacokinetic behavior of carbamazepine (CBZ), a tricyclic anticonvulsant, after its oral administration was affected by concurrent treatments with these fibers in rats 7,8. Therefore, we hypothesized that a pharmacokinetic interaction may occur between CBZ and semi-solid enteral nutrients.

The aim of the present study was to investigate whether the pharmacokinetic profile of orally administered CBZ was altered in rats by concurrent treatments with semi-solid RACOL^®^ and HINE E-gel^®^, which are semi-solid enteral nutrients marketed in Japan.

## Materials and Methods

### Chemicals

CBZ was purchased from Wako Pure Chemical Ind. (Osaka, Japan). Liquid and semi-solid RACOL^®^ and HINE E-gel^®^ were obtained from Otsuka Pharmaceutical Co., Ltd. (Tokyo, Japan). All other reagents were of commercial or analytical grade, requiring no further purification.

### Animal care and treatment

Male Sprague-Dawley rats, aged 7 weeks, were obtained from Japan SLC, Inc. (Hamamatsu, Japan). Rats were acclimatized for at least 2 days before being assigned to their experimental groups, and were housed in a clean room maintained at 23 ± 2°C with a relative humidity of 55 ± 10% and 12-h light/dark cycle. They were allowed free access to a regular animal diet and tap water. The left jugular vein of rats was cannulated with polyethylene tubing (Natsume Seisakusyo Co., Ltd., Tokyo, Japan) under anesthesia, and the tube was then externalized to the interscapular area. Rats were fasted overnight following surgery. CBZ was orally administered at a dosage of 75 mg/kg and volume of 2.5 mL/kg. Each semi-solid enteral nutrient at a dosage of 2.5 kcal/kg was orally administered to rats immediately after CBZ dosing. Liquid and semi-solid formulations are both marketed in Japan for RACOL^®^, and thus liquid RACOL^®^ (2.5 kcal/kg) was orally administered to control rats under the same conditions as the semi-solid preparations. Serial blood samples were obtained from the left jugular vein 2, 5, 15, and 30 min, and 1, 2, 3, and 5 hr after the oral administration of CBZ and were replaced with an equal volume of saline. In order to maintain patency, a small volume of heparinized saline was used to fill the cannula after the collection of each blood sample. Heparinized saline was removed just before the collection of the next blood sample. Collected blood was centrifuged at 3,000 *g* for 10 min to obtain serum samples. The experimental protocols and animal care methods used in the present study were approved by the Animal Experiment Committee at Osaka Ohtani University.

### Measurement of serum CBZ concentrations

Fifty microliters of 50 μg/mL phenacetin, an internal standard, 50 μL of 0.1 M sodium hydroxide, and 750 μL of ethyl acetate were added to a 100-μL serum sample. The mixture was then vortexed and centrifuged at 5,000 *g* for 5 min. The organic layer was decanted into new tubes and evaporated using a centrifugal concentrator for dryness. The residues were resolved in 200 μL of the mobile phase and 50 μL was injected into the HPLC system (Shimadzu, Kyoto, Japan). The mobile phase consisted of 15 mM potassium phosphate buffer (pH 4.0) and acetonitrile (v/v: 66:34), and the flow rate was set at 1.0 mL/min. Absorbance of the eluent was monitored at 220 nm. The value of the area under the curve (AUC_0→5h_) was calculated by the linear trapezoidal method.

### Statistical analysis

Data were expressed as means ± S.D. Comparisons among groups were made using an analysis of variance (ANOVA) followed by Tukey's test. Differences with a *p*-value of 0.05 or less were considered to be significant.

## Results

### Pharmacokinetics of CBZ after its oral administration

**Figure 1:**
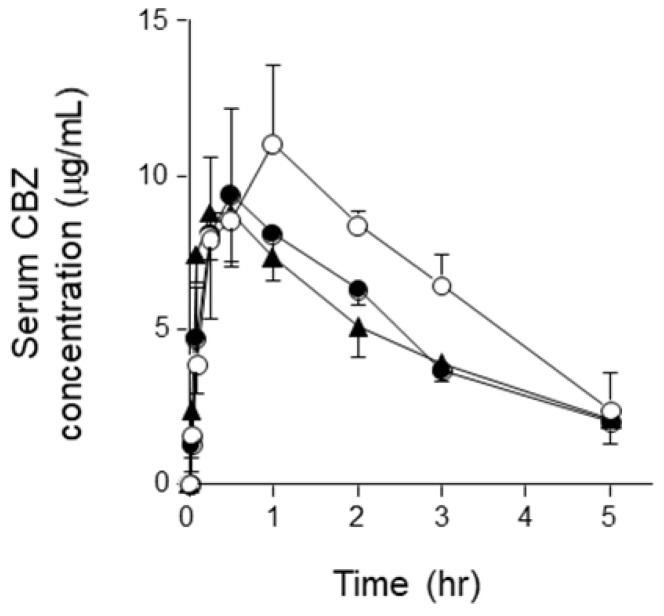
Serum concentration-time courses of CBZ after its oral administration to rats. Serum concentrations of CBZ were measured after its oral administration (75 mg/kg). Results are shown as the means ± SD of four rats per group. Open circle: the liquid RACOL^®^ group; Closed circle: the semi-solid RACOL^®^ group; Closed triangle: the HINE E-gel^®^ group.

**Figure 2:**
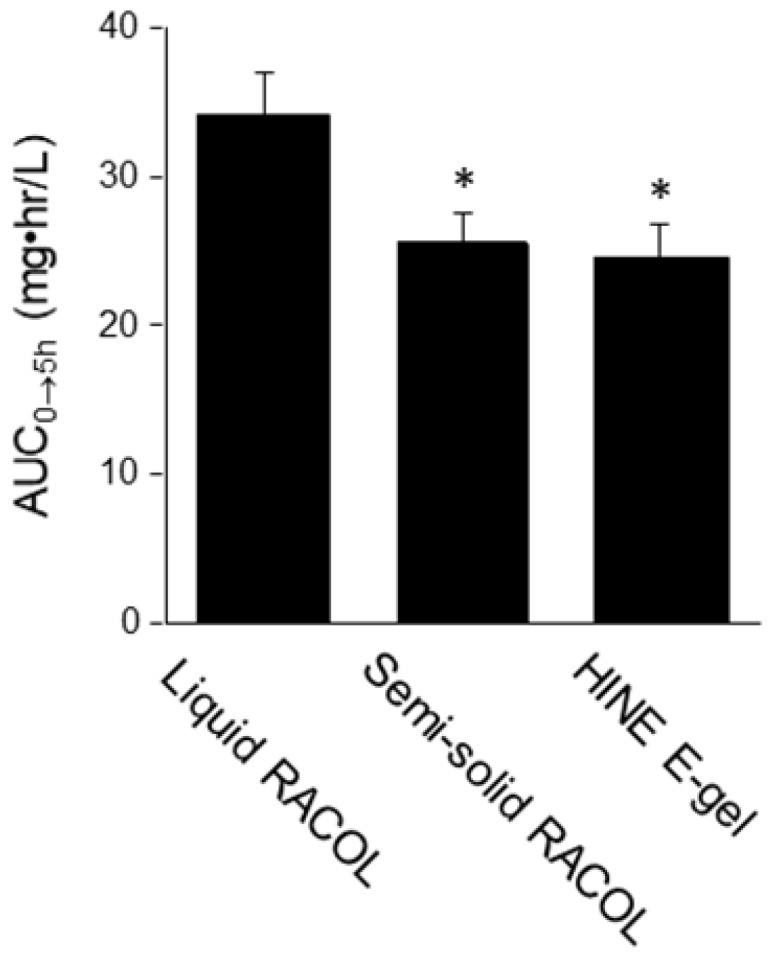
AUC_0→5h_ values of CBZ after its oral administration. The values of AUC_0→5h_ were calculated by the linear trapezoidal method. Results are shown as the means ± SD of four samples per group. *: Significantly different from the mean value of the liquid RACOL^®^ group.

The serum concentration-time profile of orally administered CBZ is shown in Figure 1. The serum concentration of CBZ at each sampling point was lower in the semi-solid RACOL^®^-treated group than in the liquid RACOL^®^-treated group. No significant differences were observed in the serum concentration of CBZ at each sampling point between the semi-solid RACOL^®^-treated and HINE E-gel^®^-treated groups. The values of AUC_0→5h_ were estimated using the linear trapezoidal method (Fig. 2). The value of AUC was significantly lower in the semi-solid RACOL^®^- and HINE E-gel^®^-treated groups than in the liquid RACOL^®^-treated group.

## Discussion

Appropriate nutrition management plays an essential role in improving clinical outcomes. The semi-solidification of enteral nutrients is available as a strategy to prevent the complications associated with liquid enteral nutrients. However, limited information is currently available on the adverse effects of semi-solid enteral nutrients. In the present study, we investigated whether the pharmacokinetic behavior of CBZ was altered in rats treated with semi-solid enteral nutrients. To the best of our knowledge, this is the first study to provide experimental evidence for a possible interaction between CBZ and semi-solid enteral nutrients.

The serum concentration of CBZ at each sampling point was lower in rats treated with semi-solid RACOL^®^ at a dosage of 2.5 kcal/kg than in those treated with liquid RACOL^®^ (2.5 kcal/kg), resulting in a decrease in the AUC value. The bioavailability of CBZ was previously reported to be primarily influenced by the extent of absorption 9. Although absolute bioavailability was not examined in the present study, relative bioavailability was estimated by comparing AUC values. Guar gum, xanthan gum, and sodium alginate, which are water-soluble fibers, are components of semi-solid RACOL^®^ that semi-solidify the formulation. We previously reported that CBZ was adsorbed by guar gum and xanthan gum in solution, which reflected gastric juice and fluid in the intestinal tract, and this may be responsible for the alterations observed in the pharmacokinetic profile of CBZ 7. Our previous findings also demonstrated the adsorption of CBZ onto sodium alginate in solution, which reflected fluid in the intestinal tract, resulting in reductions in the serum levels of CBZ following its oral administration 8. Furthermore, SA was found to gel in the stomach when ingested 10. Gelled SA was considered to have interacted with CBZ and affected the dissolution process of CBZ. Therefore, we suggest that the pharmacokinetic behavior of orally administered CBZ differed when administered concurrently with liquid RACOL^®^ and semi-solid RACOL^®^, and also that the serum concentration of CBZ was reduced by the semi-solidification of enteral nutrients through the absorption process. Serum concentrations of CBZ at each sampling point after its oral administration to rats treated with HINE E-gel^®^ were similar to those obtained in rats treated with semi-solid RACOL^®^, which was reflected in the value of AUC. Pectin and calcium ions are components of HINE E-gel^®^. Divalent metal ions, such as calcium ions, crosslink between free carboxyl groups in pectin molecules to form a network structure, which results in the gelation of pectin. Gelled pectin was considered to have interacted with CBZ and affected the dissolution process of the drug. Therefore, we suggest that the pharmacokinetic behavior of CBZ was affected by a simultaneous treatment with HINE E-gel^®^ similar to that with semi-solid RACOL^®^.

There is currently no clinical evidence for a pharmacokinetic interaction between CBZ and semi-solid enteral nutrients. However, the proposed daily requirement of nutrition for Japanese adults is approximately 20-25 kcal/kg to maintain the basal metabolic rate, which is markedly higher than the dose adopted in the present study (2.5 kcal/kg). Thus, the pharmacokinetic behavior of CBZ may be altered by a treatment with semi-solid enteral nutrients in clinical practice, necessitating healthcare personnel to pay careful attention to unexpected therapeutic failures. The pharmacokinetics of CBZ in regular diet may be close to that in semi-solid enteral formula, since the diet contains abundant dietary fiber. However, the fibers contained in the regular diet and the enteral nutrients are different, and the fibers contained in the latter have the property of strongly adsorbing to the drug. Therefore, it was thought that patients taking these semi-solid enteral nutrients had lower blood levels of CBZ than patients taking their diet, which in turn reduced the therapeutic efficacy of CBZ. On the other hand, individual differences may exist in the alterations that occur in serum levels of CBZ by treatments with semi-solid enteral nutrients because the dissolution rate of CBZ in gastrointestinal fluid was found to be slow and the drugs possessed anticholinergic properties (Chen et al., 2002). Furthermore, tablets and capsules are widely used in clinical practice, although some patients are administered by the simple suspension method, and thus their disintegration and dissolution are extremely important factors when evaluating pharmacokinetic interactions. In this regard, further clinical examinations on the interaction between CBZ and semi-solid enteral nutrients are needed in the near future.

In conclusion, we herein demonstrated using rats that a pharmacokinetic interaction may occur between CBZ and semi-solid enteral nutrients, such as semi-solid RACOL^®^ and HINE E-gel^®^. Our results will contribute to promoting appropriate nutritional management in pharmacotherapy.
